# Diagnosing Microcystin Intoxication of Canines: Clinicopathological Indications, Pathological Characteristics, and Analytical Detection in Postmortem and Antemortem Samples

**DOI:** 10.3390/toxins11080456

**Published:** 2019-08-03

**Authors:** Amanda J. Foss, Mark T. Aubel, Brandi Gallagher, Nancy Mettee, Amanda Miller, Susan B. Fogelson

**Affiliations:** 1 GreenWater Laboratories, Palatka, FL 32177, USA; 2 Animal Care Cancer Clinic, Stuart, FL 34994, USA; 3 Pet Emergency of Martin County, Stuart, FL 34994, USA; 4 Fishhead Labs LLC, Stuart, FL 34997, USA

**Keywords:** HAB, microcystin, Adda, canine intoxication, MMPB, urinalysis, hair, ELISA, LC-MS/MS

## Abstract

In the summer of 2018, six dogs exposed to a harmful algal bloom (HAB) of *Microcystis* in Martin County Florida (USA) developed clinicopathological signs of microcystin (MC) intoxication (i.e., acute vomiting, diarrhea, severe thrombocytopenia, elevated alanine aminotransferase, hemorrhage). Successful supportive veterinary care was provided and led to survival of all but one patient. Confirmation of MC intoxication was made through interpretation of clinicopathological abnormalities, pathological examination of tissues, microscopy (vomitus), and analytical MC testing of antemortem/postmortem samples (vomitus, blood, urine, bile, liver, kidney, hair). Gross and microscopic examination of the deceased patient confirmed massive hepatic necrosis, mild multifocal renal tubular necrosis, and hemorrhage within multiple organ systems. Microscopy of a vomitus sample confirmed the presence of *Microcystis*. Three analytical MC testing approaches were used, including the MMPB (2-methyl-3-methoxy-4-phenylbutyric acid) technique, targeted congener analysis (e.g., liquid chromatography tandem-mass spectrometry of MC-LR), and enzyme-linked immunosorbent assay (ELISA). Total Adda MCs (as MMPB) were confirmed in the liver, bile, kidney, urine, and blood of the deceased dog. Urinalysis (MMPB) of one surviving dog showed a high level of MCs (32,000 ng mL^−1^) 1-day post exposure, with MCs detectable >2 months post exposure. Furthermore, hair from a surviving dog was positive for MMPB, illustrating another testable route of MC elimination in canines. The described cases represent the first use of urine as an antemortem, non-invasive specimen to diagnose microcystin toxicosis. Antemortem diagnostic testing to confirm MC intoxication cases, whether acute or chronic, is crucial for providing optimal supportive care and mitigating MC exposure.

## 1. Introduction

Harmful algal blooms (HABs) resulting in animal intoxications are a worldwide occurrence, with reports of mortality becoming more prevalent [1,2]. Shoreline cyanobacteria blooms are one type of HAB that can lead to exposure of those living near lakes and streams, such as domestic dogs. Dogs represent a sentinel species due to their shared environment with humans [3], supporting the framework for a One Health approach when investigating cyanotoxins. Cyanobacteria poisoning of canines has been well documented, but comprehensive reporting on toxin levels detected in specimens is sparse [2,4,5,6,7]. Reports of canine intoxication by cyanobacterial neurotoxins are more prevalent, but intoxication to hepatotoxins such as microcystin (MC) have increased in frequency [2]. Over a four-year period (2007–2011), Departments of Health and/or Environment from 13 states reported 43 dogs suspected of poisoning by MCs with a moderate to high probability, based on clinical and diagnostic pathology [2]. Awareness of these events is spreading; however, MC intoxication events likely go under reported. This could be due to a multitude of contributing factors, such as; insufficient exposure history, lack of supportive environmental data, lack of standard HAB protocols or policies leading to inadequate sample acquisition and/or handling, improper analytical test selection, or misdiagnosis due to commonality of symptoms to other hepatotoxins. Furthermore, monetary restrictions may hinder testing beyond preliminary veterinary intervention. Therefore, providing veterinarians and analytical laboratories information on proper specimen collection protocols is of the utmost importance. Protocol dissemination will help to minimize costs, provide clinically relevant information, and compile data to inform the community of local environmental threats.

Typical MC canine toxicosis cases are the result of cyanobacteria ingestion. Post ingestion, it has been shown that MCs make their way through the gastrointestinal tract and into the liver, presumably through first pass effects through the bile-acid transport system [8,9,10]. MCs do not passively enter cells, but require active transport [11], mediated by organic anion transporters (OATs) [12]. These transporters are not only significantly expressed in the liver (Oatp1b2, OATP1A2, OATP1B1, OATP1B3) [12], but are also expressed in the brain and kidney (OATP1A2) [12,13]. The OAT facilitated uptake of MCs is one of the key steps in the pathogenesis of the reported hepatocellular damage and may account for the neurological effects observed in animals and humans following exposure [14,15]. The predominant OATs in the liver and kidney of canines (Oatp1b4 > Oatp2b1 > Oatp1a2) appear to exhibit similar substrate specificity to that of the human OATP1B3 [16]. Human OATP1B1 is abundant in lobular hepatocytes, while OATP1B3 is predominantly expressed in hepatocytes near the central vein [17], indicating interspecies differences in OAT location may play a role in clinical presentation of toxicosis. Once in the cytoplasm, MCs can affect a variety of cellular pathways, including regulation of DNA repair, regulation of protein activity, cell signaling, cell cycle, gene expression, apoptosis, and metabolism of endogenous or cytotoxic compounds [18]. The most studied of these pathways is the inhibition of the essential members of the protein phosphatase (PP) family. The reversible phosphorylation of proteins is an integral part of metabolism, which MCs inhibit by binding to serine/threonine PP1, PP2A [19] and PP3 [20]. PP inhibition can result in hyperphosphorylation [21], increases in reactive oxygen species (ROS) [22] and/or inflammation. These effects result in the disruption of cytoskeletal components, rearrangement of actin filaments within hepatocytes, and ultimately cellular death, which elucidates the observed morphological changes post-mortem [23].

The main pathway of hepatic elimination of MCs is Phase II biotransformation through conjugation with glutathione (via glutathione-S-transferase or non-enzymatically) [21,24,25,26] and through an elimination conjugation reaction with cysteine [24]. In mammals, MCs are primarily eliminated by both biliary and renal routes, with conjugated forms excreting mainly through the kidneys [27,28,29]. Since MC conjugates retain some of their toxic potential [30], metabolites may lead to continued insult to vital organ systems. The extent of intoxication and ability to recover from MC exposure is dependent on dose and the animal’s capability to metabolize MCs. Since the antioxidant glutathione is integral to the detoxification and elimination of MCs, depletion after a high dose or in the presence of concomitant contaminants has been observed [31]. The loss of active glutathione coupled with continued hyperphosphorylation and resultant ROS formation likely contributes to the necrosis and apoptosis of hepatocytes, as well as the breakdown of the hepatocyte cytoskeleton. As the primary site of MC detoxification, the hepatic parenchyma exhibits the most striking damage to intoxication; however, the renal nephron can also be negatively affected [5,32,33]. Although the cause of renal parenchymal damage has not been identified, tubular ischemia has been proposed as one mechanism [5]. Other probable mechanisms include hepatic shock and direct toxic action to the renal tubules of conjugated and free MCs.

The proper analytical approach is key to confirmation of MC exposure. Advances in MC research have elucidated numerous structurally related congeners, which have increased from 60+ known in the 1990’s [34] to over 250 described to date [35]. Variations along the structure occur mainly in two amino acid positions (X^2^, Y^4^; Figure S1), but modifications, such as desmethylation, may happen along other parts of the structure. This structural variation coupled with protein binding and conjugate formation present significant analytical challenges. Therefore, widely available commercial enzyme-linked immunosorbent assays (ELISAs) are frequently used to test MCs due to their broad specificity to the various congeners (and potentially conjugates). However, MC ELISAs have a narrow range of applicability to complex matrix testing (e.g., urine, tissue, blood) from different animal species, with false positive results reported when analyzing mammalian livers [36]. While their availability and ease of use make them convenient, they also require an alternate method of confirmation, such as liquid chromatography tandem-mass spectrometry (LC-MS/MS). The specificity achieved when targeting MCs via LC-MS/MS provides quantitative accuracy, but also results in the under-reporting of total MCs [37,38]. This is due to a lack of commercially available reference materials for method calibration coupled with the extensive variability in MC forms. Other techniques utilized to address this include non-targeted high- and low-resolution LC-MS, but this requires an intimate knowledge of MC chemistry. An alternate approach involves the oxidative cleavage of the unique Adda (3-amino-9-methoxy-2,6,8-trimethyl-10-phenyl-4,6-decadienoic acid) side chain and subsequent quantitative analysis of MMPB (2-methyl-3-methoxy-4-phenylbutyric acid). The MMPB technique provides a relatively straightforward protocol accounting for total Adda containing MCs or nodularins [38,39,40]. This test allows for the quantification of free MCs and those modified during metabolism as long as there is conservation of the Adda side chain.

At present, the diagnosis of cyanobacteria poisoning requires a thorough history of the exposed patient in relation to the contaminated source. A two-tier analysis should include identification of the dominant cyanobacteria genera present and toxin analyses. In the absence of an algal grab sample, analyses can be conducted on the vomitus/stomach contents of a recently exposed individual, which is representative of unmetabolized cyanotoxins. However, due to the low pH of gastric contents, degradation of the organisms may impede identification of cyanobacteria genera. Thus, broad screening of multiple cyanobacterial toxins or targeted analysis for specific toxins based on clinicopathological data may be required. Once toxins are confirmed in the source, additional targeted analyses should be conducted on other specimens (e.g., liver, kidneys, feces, and urine) to confirm exposure and metabolism. Data achieved from the source of exposure, coupled with clinical/pathological observations and analytical data have been utilized to confirm MC intoxication in previous studies [4,5,6]. This process is time consuming, cost prohibitive, requires significant knowledge of cyanobacteria and the toxins they produce, and many specimens are not ideal for antemortem testing. Therefore, a standard protocol for diagnosing MC toxicosis should be developed for non-invasive specimen collection and sensitive accurate testing.

The present study illustrates the most comprehensive report on the pertinent clinicopathological data, pathological characteristics, supportive care, and novel diagnostic testing performed during and after an exposure event involving dogs. Results from this investigation provide support of viable antemortem testing methods for detection of MCs in canines during and after suspected exposure to microcystin producing cyanobacteria.

## 2. Results

### 2.1. Presentation, Clinical Data and Treatment

Between 26 August to 8 September 2018, six dogs were admitted for medical care at Pet Emergency of Martin County, Florida, USA. Information pertaining to the exposed animals and negative controls used in this study are presented in Table 1. The patient history for the six hospitalized cases included access to the Indian River and potential ingestion of decaying fish, organic debris, or water from the waterway. The onset of clinical signs varied from 2–48 h post exposure, with the most common signs being vomiting and depression. Weakness, collapse, tachycardia, petechia/ecchymosis and melena were also noted in a subset of patients. Clinicopathological findings included but were not limited to: elevated ALT (Alanine aminotransferase), thrombocytopenia, prolonged partial thromboplastin time (PTT) and prothrombin time (PT), peritoneal and/or pleural effusion, hypoglycemia and hyperbilirubinemia. For a full list of abnormalities and values, refer to Table 2.

After presentation to the emergency clinic, a complete blood count, blood chemistry, and clotting profiles were performed. Decontamination through bathing was initiated in a subset of patients prior to arrival. One patient presented with productive emesis and vomitus was saved for cyanotoxin evaluation. The Animal Poison Control Center was contacted for advice on the cases but ultimately treatment was tailored for each dog by the attending veterinarian with the emphasis on acute liver injury. Therapy included intravenous fluids (with dextrose supplementation as indicated), gastroprotectants, antibiotics, antiemetics, analgesics, fresh frozen plasma (FFP), cholestyramine, vitamin K, N-acetylcysteine and various oral liver protectants. For the dogs that required intensive overnight care, continuous monitoring of electrocardiogram and blood pressure were performed. Furthermore, other essential parameters were monitored at varying intervals such as blood glucose, electrolytes, activated partial thromboplastin time/prothrombin time (APPT/PT), complete blood count (CBC), and blood chemistry. Patient hospitalization ranged from one day to nine days.

### 2.2. Pathology

One of the six dogs succumbed to fulminant liver failure, coagulopathy, and shock. A full postmortem examination was performed (Figure S2). On preliminary gross examination, the dog was in good body condition with a body condition score of 5/9 and mild post-mortem autolysis [41]. The skin in areas without hair appeared slightly yellow with multifocal areas of ecchymosis and petechiation. Diffuse icterus of the mucous membranes and subcutaneous adipose tissues was noted. Abundant dark red to black fluid drained from the nares and oral cavity upon manipulation of the head. Entry into the abdominal cavity showed up to 1L of serous red tinged fluid. The length of the intestines was dark pink to red with red streaking down the serosa, which had a granular appearance. Abundant edema and coagulated blood expanded the mesentery and omentum adjacent to the spleen as well as around the pancreas. Inspection of the esophagus, stomach, and intestines revealed abundant dark red to black fluid that filled the entire gastrointestinal tract. The gastric wall at the pylorus was diffusely expanded by submucosal hemorrhage and edema. The liver was diffusely dark red with sharp margins, a reticular pattern, and had a normal consistency. The gall bladder was filled with dark green bile and the wall of the bladder was thickened by edema. Abundant bright yellow, granular thick fluid was present in the urinary bladder. However, the kidneys and ureters were intact. The spleen was diffusely pale red and had multiple <3 mm fibrotic nodules on the serosal surface. The right lung lobes appeared pink except for a 3–4 cm red focus in the cranial lobe and mild dark red mottling in the middle lobe. The left lobes were diffusely red, wet, and oozed abundant red fluid upon transection. The adrenal glands had bilateral hyperplasia of the cortical layers. Multifocal petechia and ecchymosis were observed in the wall of the great vessels of the heart and in the endocardium of the left ventricle. There was mild multifocal, nodular thickening of the mitral valve leaflets.

**Figure 1:**
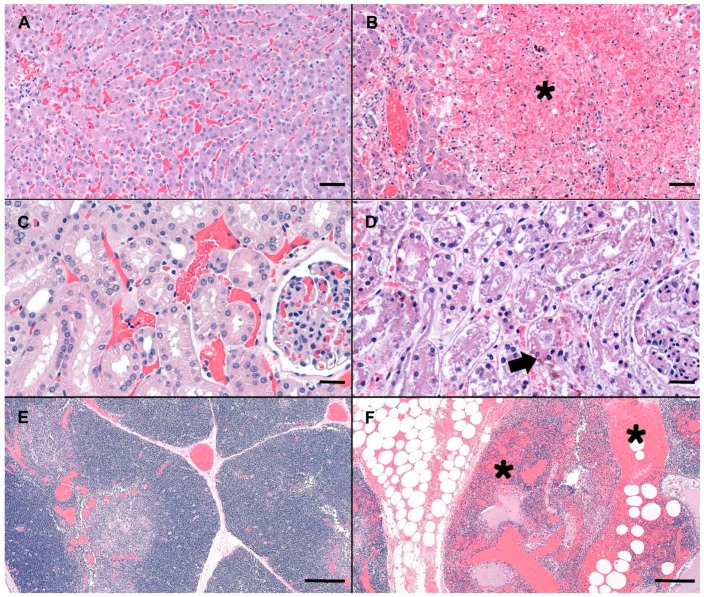
Photomicrographs (canine) of hematoxylin and eosin stained (H&E) normal liver, renal cortex, and thymus (**A**,**C**,**E**) as compared to the MC exposed dog (C-SP) (**B**,**D**,**F**). (**B**) Severely disrupted hepatic cords characterized by massive hepatocellular necrosis and hemorrhage (asterisk). Low numbers of hepatocytes adjacent to a central vein are spared. (**D**) Renal cortex with a locally extensive area of acute tubular necrosis. Note accumulation of brown granular pigment within tubular epithelial cytoplasm or sloughed cellular debris within the tubular lumina (arrow). (**F**) Mediastinal adipose and thymus expanded by hemorrhage, fibrin and edema (asterisks). Scale bars are 100, 50, and 500 µm for liver, renal cortex and thymus, respectively.

Compared to normal canine microscopic anatomy (Figure 1A,C,E), tissues of the deceased dog showed several significant microscopic changes including; acute, severe, massive hepatocellular necrosis (Figure 1B), acute, moderate, multifocal, tubular necrosis with granular casts and intracellular iron (Figure 1D), acute, severe, multifocal to coalescing, hemorrhage in the thymus, gastrointestinal tract, mesentery, omentum, lymph nodes, pancreas, lungs, pulmonary artery, endocardium (Figure 1F), acute, diffuse, gall bladder edema, and acute, diffuse, splenic contraction with multifocal siderofibrotic plaques. Mild mitral valve endocardiosis was also noted in this patient as an incidental finding.

Ancillary testing included aerobic culture of the liver and leptospirosis PCR. Results of the culture revealed bacterial organisms *Enterococcus faecalis* and *Erysipelothrix rhusiopathiae.* Leptospirosis PCR was negative.

### 2.3. Phycology

**Figure 2:**
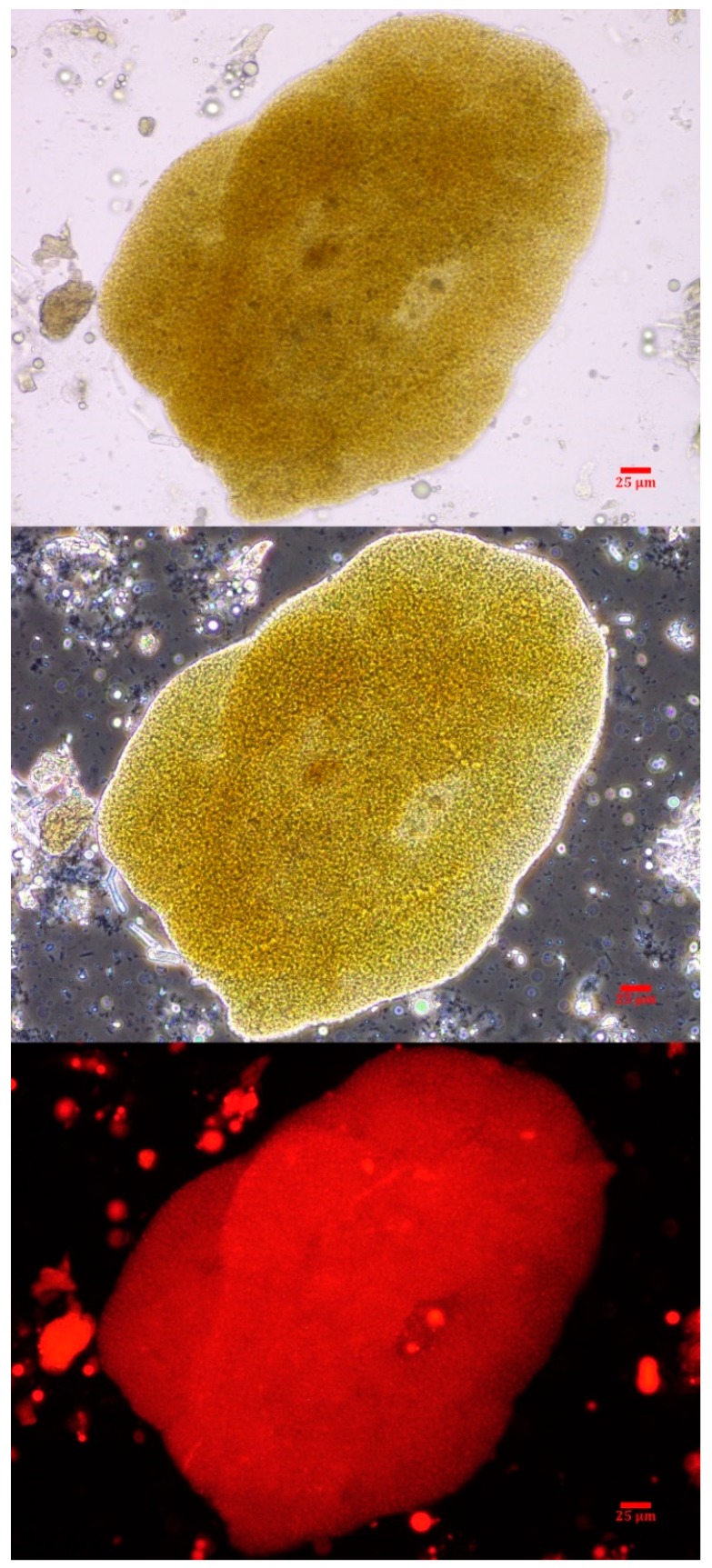
A *Microcystis* colony observed in the canine vomitus sample acquired within 6 h of exposure. The micrographs are at 400× with brightfield (top), phase-contrast (middle) and epi-fluorescence (bottom). The scale bar represents 25 µm.

Intact colonies of *Microcystis* were observed in the canine vomitus sample, confirming exposure to cyanobacteria (Figure 2). Other cyanobacteria were not observed. While a water sample was not submitted in conjunction with this exposure event, reports from the Florida Department of Environmental Protection (FDEP) support the vomitus phycological observations match the dominant genera (*Microcystis*) present in the St. Lucie River HAB at the time of exposure [42].

### 2.4. Adda Microcystin/Nodularin (MC/NOD) Levels

**Figure 3:**
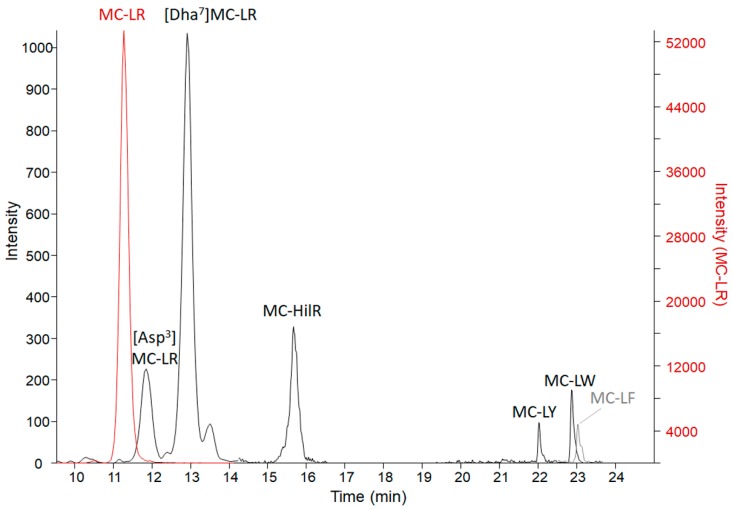
The LC-MS/MS chromatograms of MC variants confirmed present in the C-GR#2 vomit sample with a sum of 14,000 ng g^−1^ MCs. The MC-LR scale is on the right due to high levels detected in comparison to the other variants. MC-LR > [Dha^7^]MC-LR > MC-HilR > [DAsp^3^]MC-LR > MC-LY > MC-LW > MC-LF. Transitions monitored are reported in Table S1.

Results from the three microcystin/nodularin (MC/NOD) analytical tests on all specimens are reported in Table 3. Enzyme-linked immunosorbent assay (ELISA) values (representing freely extractable Adda MCs/NODs) were, in general, supported by the MMPB (2-methyl-3-methoxy -4-phenylbutyric acid) data (representing total Adda MCs/NODs). This indicates that the Adda ELISA is able to react to conjugated forms of microcystin, as those excreted in urine. However, total Adda MCs (measured as MMPB) were higher in organ specimens, supporting that the MMPB method accounts for some fraction of protein bound MCs. The only non-metabolized specimen, the vomitus, had total Adda MCs (MMPB) measured at 46,000 ± 8000 ng g^−1^, confirming a high dose of exposure. The vomitus sample was significantly diluted with meal items (>30 grams submitted), so the dose was in excess of 1,380,000 ng total MCs. Analysis of the vomit using ELISA resulted in a lower level of MCs (25,000 ± 1800 ng g^−1^), which may be due to MC losses to the matrix (high protein dietary items), partial MC degradation or due to differences in analytical technique used. Targeted LC-MS/MS of 19 MC variants and NOD-R confirmed the presence of 7 MC variants (Figure 3) and the absence of NOD-R. The dominant variant present was MC-LR (14,000 ± 100 ng g^−1^), followed by [Dha^7^]MC-LR (170 ± 21 ng g^−1^), MC-HilR (140 ± 28 ng g^−1^), [Asp^3^]MC-LR (82 ± 0 ng g^−1^), MC-LY (23 ± 16 ng g^−1^), MC-LW (18 ± 3 ng g^−1^), and MC-LF (14 ± 2 ng g^−1^). Other variants were detected in a non-targeted MS scan, but due to a lack of standards available to verify identities or levels, they are not reported here (work ongoing). Targeted MCs in the vomit accounted for 56% of the ELISA measurement and 30% of the total Adda MCs by MMPB. The results of the targeted MC analysis were key to the decision to target MC-LR in the remaining collected specimens.

MC-LR was detected in all the tested specimens from exposed animals with exception of the hair (C-GR #1) and one blood sample (C-GR #2). The negative control specimens were all below detection for targeted MC-LR. The remaining MC-LR concentrations (metabolized specimens) only accounted for 0.5%–2.8% of ELISA data and 0.3–2.3% MMPB data.

**Figure 4:**
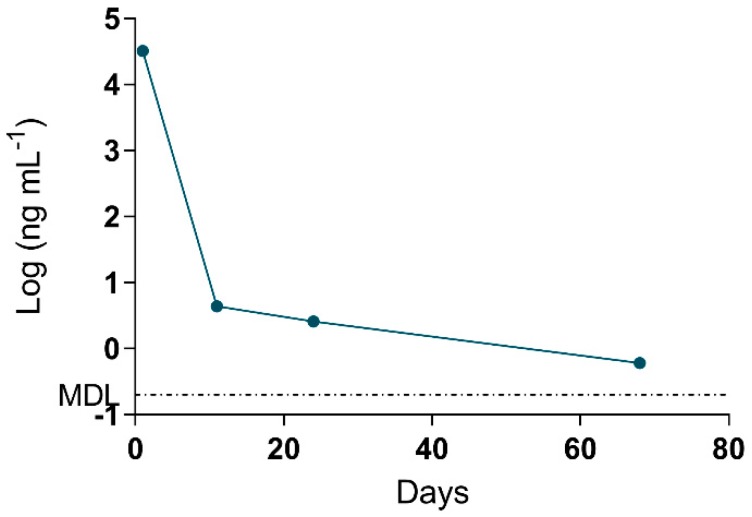
Data derived from the log of total Adda MCs (by MMPB) of the urine collected from one of the surviving dogs (C-GR#2) plotted against days post exposure. Urine was collected within 1 day from initial exposure event, and the animal continued to excrete MC metabolites >60 days post exposure. The MDL for total MCs in urine was determined to be 0.2 ng mL^−1^.

**Figure 5:**
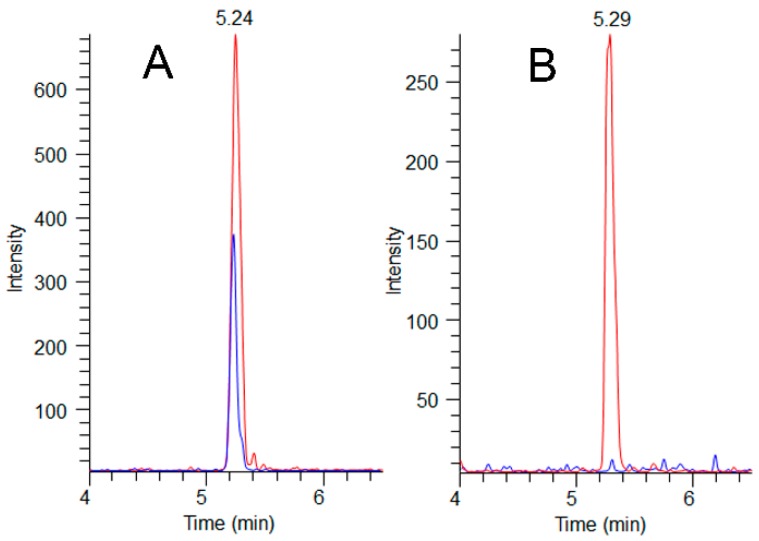
MMPB LC-MS/MS chromatograms (*m*/*z* 207→131) showing sample peaks (blue) overlaid with their paired pre-oxidation MC-LR spikes (red) at 200 ng g^−1^ for A (exposed dog C-GR#1) and 100 ng-g^−1^ for B (unexposed dog C-CBR). Total MCs detected in the C-GR#1 hair at 72 days post exposure was determined to be 180 ng g^−1^, illustrating hair as a potential route of MC elimination.

The MMPB data was integral to results interpretation, as the data is representative of total Adda MCs (and nodularins, when present), regardless of form (free, bound, partially degraded). Since the approach to quantification was pre-oxidation spiking (standard addition), confidence in MMPB data was higher than that of ELISA. The spike returns in Table 3 are shown only for reference and are used to illustrate how the method is impacted by various complex matrices (as compared to MC-LR oxidized in water). A low level of detection (sub-ppb) was achieved for urine and blood samples. The strength of this approach was illustrated when 2.6 ng mL^−1^ total MCs was detected in the urine sample of C-Pom over 3 weeks after suspected exposure. While this level could not be confirmed using alternate techniques due to higher test MDLs, the MMPB urinalysis of another exposure case (C-GR#2) supported the continued excretion of Adda >60 days post exposure (Figure 4). In addition to renal elimination, it was determined that hair was a potential route of elimination. MMPB chromatograms of a dog hair sample collected 72 days post exposure with overlaid pre-oxidation spikes of MC-LR can be viewed in Figure 5, with a negative control sample. A total MCs of 180 ng g^−1^ (dry weight) was determined to be present in the C-GR#1 hair specimen.

**Figure 6:**
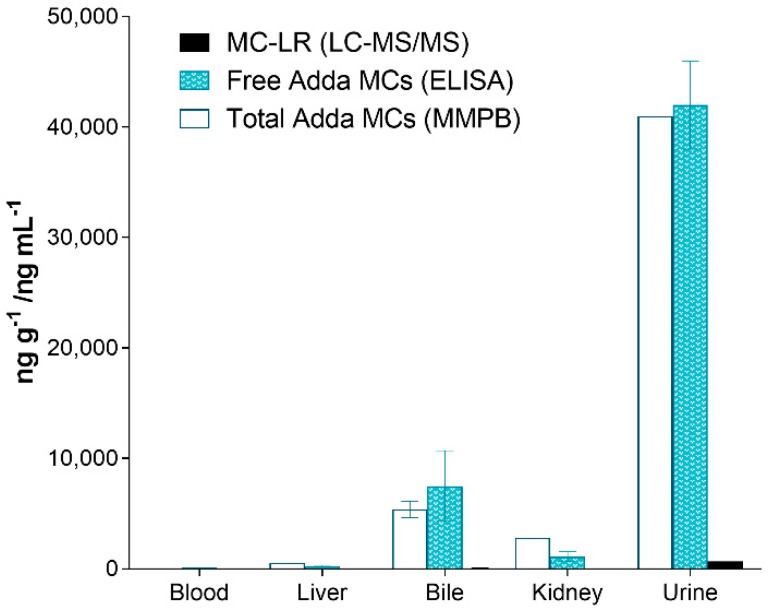
Results showing the different specimens collected from the dog (C-SP) that succumbed to intoxication 2 days post exposure and reported as ppb (ng mL^−1^ or ng g^−1^). As illustrated, the urine contained the highest amounts of MCs (all methods), followed with the bile, kidney, liver and finally heart blood. All specimens were collected post-mortem.

The testing of the organs of the deceased canine (C-SP) revealed that the kidney had higher total Adda MCs (MMPB), free MCs (ELISA) and MC-LR when compared to the liver. In similar fashion, the urine was higher than the bile, with the least MCs measured in heart blood. Regardless of method used to test the specimens, the levels of MCs were as follows: urine > bile > kidney > liver > blood. Figure 6 illustrates the MCs detected using the 3-techniques in the specimens collected from the deceased dog (C-SP).

It should be noted that the method of quantification for each technique is different, with interpretations of spike returns and final data essential to understanding results. The Adda ELISA method employs an external curve (certified reference material of MC-LR) for quantification, with the only assessable quality controls being replicate extraction data (reported as the average with standard deviations in Table 3) and spike returns. Both high (>130%) and low (<70%) spike returns were obtained from ELISA data. Exaggerated spike returns observed with ELISA may indicate overestimates of natively reported MCs, as supported by MMPB data in the bile sample. In contrast, low spike returns (e.g., hair and liver specimens), indicate matrix inhibition. The same extracts (and MC-LR spikes) were analyzed by both Adda ELISA and LC-MS/MS and can be directly compared. The MC-LR spikes analyzed by LC-MS/MS returned 57% to 111%, while the same spikes were 0% to 211% when analyzed with the ELISA, further supporting that the ELISA resulted in both exaggerated and inhibited responses.

Further complicating ELISA data interpretation was the positive (≥15 ng g^−1^ MCs/NODs) response elicited from a negative control liver sample. The negative control specimen was collected from an animal without a recent exposure to waterways or cyanobacteria. Furthermore, MMPB analysis confirmed that Adda MCs/NODs were not present over 4 ng g^−1^, which is more sensitive than the ELISA MDL of 15 ng g^−1^. Sample extract clean-up (SPE), dilution (100-fold) and hexane washing were not sufficient in mitigating false positive data for the negative control liver sample. In comparison, prior to hexane washing, the positive liver and kidney samples were 260 and 1105 ng g^−1^, respectively. Post hexane washing, the levels were similar at 258 and 1080 ng g^−1^, with little change to spike returns (193→201%, 106→111%). Hexane washing did lower the negative control liver assay responses (analyzed with 100-fold dilution) from 1.78 to 0.52 ng mL^−1^ (correlating to 178 to 52 ng g^−1^), and increased the spike return from 12% to 33%. However, this was not sufficient in preventing the false positive assay result.

## 3. Discussion

Harmful algal blooms (HABs) can be hazardous to humans, animals and the environment [43,44]. Reports of bloom formation with toxin production are becoming more frequent, likely due to anthropogenic influence leading to nutrient accumulation in waterways and changing global environmental conditions [45,46,47]. The 2018 *Microcystis* bloom in the St. Lucie River is a fitting example of the sublethal and lethal toxic effects that may be observed in dogs exposed to HABs.

The clinical signs and pathology of blue-green algal toxicity can mimic a handful of toxicoses, including, but not limited to, xylitol, sago palm, rodenticide, *Amanita*, and ricin. Rapid detection of the inciting toxin can be problematic in clinical settings. Samples of tissues may be difficult or dangerous to obtain and few laboratories provide cyanotoxin testing for animal tissues. In this work, the source water was not available for testing, but *Microcystis* and MCs were determined present in the source water during the event by the Florida Department of Environmental Protection [42]. The vomit from one of the six dogs did have measurable MCs, with MC-LR found to be 97% of the total targeted MCs. This observation is similar to other work analyzing MCs in the St. Lucie River [48], where MC-LR was 98% of the total targeted variants by LC-MS/MS. Specimens tested in past dog exposure events that succumbed to MC intoxication included the source water, feces [4], liver [5], and vomitus [5,6]. In this work, clinicopathological observations, post-mortem pathology, and MC testing were all used to confirm that the exposed dog morbidity was a result of MC intoxication and findings correlate with previous reports [4,5,6,49]. This work illustrates the most comprehensive multidisciplinary reporting on microcystin induced animal morbidity and mortality following a HAB exposure event. Confirmation of cyanobacteria intoxication is rare in a clinical setting due to a multitude of reasons. Improved antemortem methods for testing is essential for diagnosis and timely treatment recommendations, especially in the face of a multi-animal exposure event.

**Figure 7:**
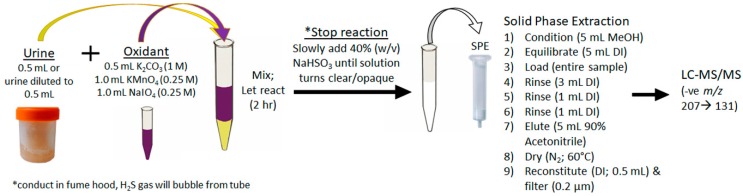
Schematic showing the recommended approach to the oxidation, extraction and clean-up of urine for the purposes of total Adda MCs by the MMPB method. In order to properly quantitate, it is essential that pre-oxidation spiking of MC-LR be conducted on duplicate sample.

For the first time, urine was used to diagnose an ongoing MC intoxication event. The importance of this finding cannot be overstated, as urine is a specimen that can be easily, quickly and non-invasively collected by veterinarians faced with a suspected toxicosis, even if several days or weeks have passed. The depuration of MCs, even months after exposure, is likely due to the accumulation of MCs in tissues and bound to proteins. The MCs are then slowly released over time, with the Adda being conserved. The achieved sensitivity and broad specificity of the MMPB test, especially for urine, was integral in confirming exposure and metabolism. Figure 7 outlines the recommended oxidation and extraction approach for screening urine samples in the event of a suspected exposure case. This approach could be applied to other sentinel species, or even following human exposure events.

With regard to more long term, chronic exposure, it has been demonstrated that hair might be a candidate for assessing exposure. This is the first use of MC testing on mammalian hair following an intoxication event and positive results were observed using the MMPB technique. This preliminary exercise shows promise with regard to potential avenues of testing in both acute and chronic mammalian exposure cases. However, confirmatory testing beyond the MMPB analysis did not detect free MC-LR in the hair above the established method detection limits. This area of research should be expanded in order to determine the applicability of this specimen for monitoring exposed populations of mammalian species.

Pharmacokinetic studies of orally ingested MCs in canines have not been conducted to date, making interpretation of measured toxin levels challenging. The amount of toxin needed to cause the observed gross and microscopic lesions has not been standardized in canine patients and extrapolation from lab animal models may not be representative. In experimental settings, the median lethal dose (LD_50_) for MCs is typically derived from an intraperitoneal (i.p.) route, which has been reported as low as 36 µg per kg body weight for mice exposed to MC-LR [50]. The oral LD_50_ of MC-LR has been reported to be as high as 5,000 µg per kg body weight for mice, and even higher for rats, supporting interspecies variability [10]. The likely presence of other protein phosphate inhibitors concomitant with MC-LR, such as other MC variants and peptides (e.g., anabaenopeptins), can also complicate interpretations of toxin data in reference to toxicity [51,52]. Since other MC variants were detected in this event, and MMPB data indicated even more variants were present but not accounted for in targeted analyses, correlating MC-LR levels from previous toxicity studies would provide little benefit. The inter-/intra-species variability combined with complicated real-world exposure, results in a high level of uncertainty with regard to MC dose and observed toxicity.

In this study, the dose for each animal was unknown, but it can be inferred from MMPB analysis of the urine samples from a surviving individual (32,000 ng mL^−1^) at 1-day post exposure and from the deceased animal (41,000 ng mL^−1^) at 2-days post exposure, that doses were similarly high and caused the observed hepatoxicity. Additionally, the single collected vomit sample provided evidence that >46 µg kg^−1^ total Adda MCs was initially ingested, but it is impossible to determine what level entered metabolism. Post metabolism, several potential MC metabolites were observed in the urine employing an MS scan, but require more sophisticated analyses, such as high-resolution mass spectrometry and deconjugation experiments, to elucidate the profile. Work to identify these metabolites is ongoing.

Although the oral exposure route has been confirmed in at least one of these animals, the potential for compounded exposure through inhalation cannot be ruled out. Inhalation of MC-LR by mice has a reported LD_50_ of 43 µg kg^−1^ [53], similar to that reported for the i.p. route. The higher toxicity observed via inhalation coupled with evidence that MCs can become aerosolized during cyanobacteria blooms [54,55] may present additional health risks to those living in close proximity to an ongoing bloom. Research has shown that low dose, subchronic exposure can result in accumulation of MCs in the mammalian liver [56]. Since dogs are considered a proposed sentinel species and they intimately share the human environment, canine exposure events such as this support the importance of a pro-active approach with regard to HABs in relation to human and animal health.

Collection of appropriate specimen type and selection of optimal analytical test are of high importance for accurate diagnosis. It was illustrated that the Adda ELISA provided false positive data and exaggerated assay responses, likely due to non-specific binding to kit antibodies. This highlights the need to confirm any ELISA data, especially for the analysis of matrices more complicated than water, for which the assay was intended. Matrix effects and exaggerated ELISA responses have been observed in other work [36,40] and require, at minimum, a secondary method of confirmation when a positive assay response is observed. In this work, both LC-MS/MS of targeted MCs and total Adda MCs by MMPB were conducted in addition to the MC Adda ELISA. However, due to method specificity, targeting MC variants, such as the MC-LR targeted in this work, under-represents total MCs, both due to MC structural variability and metabolic transformations. Therefore, if only one method is to be used in initial screening of samples in a suspected exposure case, it is recommended that the MMPB method be used. The MMPB approach provided a value representative of total (e.g., bound, free, conjugated) Adda MCs for the evaluated samples with a low detection limit.

Although clinicopathological data and epidemiology supported involvement of six dogs in the described morbidity/mortality event, only four dogs were confirmed to have been exposed to MCs using analytical techniques. Due to monetary constraints and lack of published sampling/testing protocols for antemortem testing of cyanotoxin in canines, confirmation could not be made in the other three cases. In the present study, a viable test using free catch urine collection has been established. Furthermore, a set protocol for directed toxin testing is described and can hopefully be employed in similar events to quickly and accurately diagnose MC intoxication. The development of an antemortem assay using non-invasive collection techniques is sure to have a significant impact on the diagnosis, treatment, and exposure of animals to HABs.

## 4. Materials and Methods

### 4.1. Pathology and Specimen Collection

A thorough gross examination of the deceased patient was performed within 12 h of death. Throughout the procedure, aseptically collected sections of liver, kidney, lung, spleen, cerebral frontal lobe, small intestine, large intestine, gastric contents, feces, urine, bile, and, heart blood were collected for ancillary testing. Fresh tissues and fluids were individually placed into sterile bags or sterile syringes to be held in a −20 °C freezer for storage until testing could be performed.

Sections of all abdominal and thoracic organs along with brain, eyes, skin, skeletal muscle, sciatic nerve, and bone marrow were preserved in 10% neutral buffered formalin. Tissues were trimmed, placed into cassettes, and processed via routine paraffin embedding techniques. All tissues were made into 3–5 µm sections and stained with hematoxylin and eosin stain (H&E) for histological review. Microscopic review was performed by a board-certified veterinary pathologist using a Nikon eclipse 80i (Nikon, Minato, Tokyo, Japan) and photomicrographs were captured by an Accu-scope Excelis HD (Commack, NY, USA).

Antemortem sample collection from a subset of the surviving dogs (Table 1) including urine, blood, vomit, and hair was performed by owners or veterinary staff during and after the initial onset of clinical signs. Urine was collected via free catch method and stored in sterile jars or plastic collection containers without preservative. Liver, kidney, free catch urine, blood and hair samples from three control dogs not associated with the HAB event were additionally collected (one deceased and the other living). Tissue, hair, and fluid samples were chilled and/or frozen prior to overnight shipment or transfer. Sample collection dates in relation to exposure can be referenced in Table 1.

### 4.2. Phycology of Vomitus Sample

A ca 0.5 g subset of the C-GR#2 vomitus sample was suspended in 5 mL of deionized water (DI) and gently vortex mixed. Wet mounts of un-fixed sample were prepared (3x) and scanned at 100X for the presence of potentially toxigenic cyanobacteria using a Nikon TE200 inverted microscope equipped with phase contrast and epifluorescence (green light excitation, 510–560, FT580, LP590). A Nikon digital sight DS-Fi1 camera was used for micrographs. Higher magnification was used as necessary for identification and micrographs.

### 4.3. Adda MC/NOD Analyses

#### 4.3.1. Specimen Homogenization

Subsets were taken from liver samples (composited 5 subsets; ca 1 g each) and the entire kidney (2.7 g) was cut into small pieces and placed into 30 mL homogenization vials with 10 mM phosphate buffer at pH 7 (1:3). The vomitus sample was emptied into a glass beaker, mixed with a spatula and subsampled (2 subsets; 1 g each) into individual 7 mL homogenization vials with extractant solution (75% acetonitrile in 100 mM acetic acid; 1:3). Acetonitrile (HPLC grade) and glacial acetic acid (>99%) were both from Thermo Fisher Scientific (Waltham, MA, USA). Ceramic beads (2.8 mm) were added and the samples were homogenized at 5 m/s for 30 seconds (1 cycle for the vomitus and 2 cycles for the tissues) using an Omni Bead Ruptor 24 (Omni International, Inc, Kennesaw, GA, USA). Hair samples were pulverized in 30 mL vials with stainless steel 2.8 mm beads dry at 6.8 m/s (30 seconds) and transferred to glass vials as 50 mg subsets. Aliquots of kidney and liver (400 µL) were transferred to glass vials for subsequent oxidations/extractions. The vomitus vials were centrifuged (1500 g; 10 min) and supernatants retained. The pellets were vortex mixed with additional extractant (1 mL), centrifuged as before and supernatants pooled and saved for extractions.

#### 4.3.2. Total Adda MCs/NODs Oxidation, Extraction and Analysis (MMPB)

MMPB oxidations and extractions were conducted as previously described [40]. Briefly, samples were oxidized as 100 mg (liver & kidney; wet weight), 50 mg (hair; dry weight), 500–1500 µL (blood), 0.2 µL–500 µL (bile, urine) and 10 mg (vomit; wet weight) subsets. All samples were paired with at least one pre-oxidation matrix spike of certified reference material (CRM) MC-LR (National Research Council Canada; Halifax, NS, Canada) for quantification. Spikes ranged from 5 ng mL^−1^ (or ng g^−1^) to 50,000 ng mL^−1^ and were added to approximately double native peak areas (positive samples) for quantification (Table S2). Subsets of liver, hair, kidney, vomit and blood were oxidized by adding 5 mL of oxidant (0.2 M K_2_CO_3_, 0.1 M KMnO_4_ and 0.1 M NaIO_4_), while urine and bile samples were oxidized with 2.5 mL oxidant. Reactions were stopped after 2 h with the addition of 40% sodium bisulfite (w/v) until solutions turned white/opaque. The liver, kidney, hair, and blood oxidized aliquots were cooled (10 min; −20 °C), centrifuged (1500 *g*; 10 min) and supernatants retained. The pellets were rinsed with DI (2 mL) by vortex mixing followed by centrifugation (1500× *g*; 10 min). Extracts were loaded onto preconditioned (MeOH→DI) 200 mg Strata X SPE (Phenomenex, Torrance, CA, USA), rinsed (DI; 3x) and eluted (5 mL; 90% acetonitrile). Extracts were blown to dryness (N_2_, 40 °C) and samples (with exception to liver and blood) reconstituted in DI (0.5–1.0 mL). The liver and blood were reconstituted in 0.1 M HCl (1.5 mL) and loaded onto simplified liquid extraction (SLE) columns (12cc; Phenomenex, Torrance, CA, USA), allowed to sit (10 min) and eluted with 2x–5mL ethyl acetate. Elutions from SLE were blown to dryness (N_2_, 40 °C) and reconstituted in DI (0.5–1.0 mL). All samples were filtered using 0.2 µm polyvinylidene difluoride (PVDF) syringe filters (Millipore Sigma, St. Louis MO, USA).

A Thermo Surveyor high performance liquid chromatography (HPLC) system coupled to a TSQ Quantum Access MAX Triple quadrupole mass spectrometer system coupled with a Kinetex C18 column (2.6 µm; 100 Å; 150 × 2.1 mm; Phenomenex; Torrance, CA, USA) were utilized as described previously [36]. The [M−H]^−^ ion of MMPB (*m*/*z* 207) was fragmented and *m*/*z* 131 (CE = 12%) was monitored. A five-point standard curve (0.5–100 ng/mL of oxidized MC-LR) was used to interpolate spike returns. Quantification of samples was conducted using individually prepared pre-oxidation spikes of MC-LR (standard addition). The instrument detection limit coupled with spike responses were used to determine the method detection limits (MDLs; Table 4).

#### 4.3.3. Free Adda MCs/NODs Extraction and Analyses

Samples with total MCs >15 ppb (ng g^−1^; ng mL^−1^) via MMPB analysis and representative negative control samples (unexposed individuals) were extracted and analyzed for free MCs, unless exhausted. Subsets of liver (100 mg), kidney (100 mg), blood/serum (500 µL), and hair (50 mg) were suspended in 5 mL extractant (75% acidified acetonitrile in 100 mM acetic acid) and sonicated (bath, 25 min). The samples were cooled (10 min; −20 °C), centrifuged (1500× *g*; 10 min) and supernatants retained. The pellets were resuspended in extractant (1 mL), cooled (10 min; −20 °C) and re-centrifuged. The pooled supernatants (including previously homogenized vomitus) were diluted (70 mL DI). Samples of urine (ranging from 10 µL–500 µL) and bile (10 µL) were diluted (10 mL DI) prior to SPE. Preconditioned Oasis HLB (200 mg; Waters Corporation, Milford, MA) or Strata X were loaded with diluted sample, rinsed with DI (2×; 5 mL and 3 mL) and eluted (5 mL; 90% acetonitrile). Solutions were blown to dryness (N_2_; 60 °C) and reconstituted in DI at sample concentrations within range of the calibration curves for each analysis used. All samples were filtered using 0.2 µm PVDF prior to analysis.

Final liver and kidney extracts were washed 1:1 (*v*/*v*) using hexane (ACS grade; Fisher Scientific, Waltham, MA, USA). Hexane was added to sample, vortex mixed, centrifuged (1500× *g*; 5 min) and the hexane layer discarded. Analysis on these extracts by ELISA was conducted both prior to and after hexane washing.

An MCs/NODs Adda ELISA (Abraxis; Warminster, PA) was used as previously described [40]. Dilutions were prepared using DI to achieve absorbance values within the range of the standard curve (0.15–4.0 ng mL^−1^) and all solutions were analyzed in duplicate. The minimum method detection limit (MDL) was 15 ppb (ng g^−1^/ng mL^−1^) based on the 100-fold dilution factor (DF) used and assay sensitivity (0.15 ng mL^−1^).

Extracts prepared for ELISA were post-spiked with the internal standard (IS) *d*_7_-MC-LR. The vomitus sample extracts were spiked with a second IS (*d5*-MC-LF). Both IS were acquired from Abraxis Kits (Warminster, PA, USA). A targeted analysis for MC-LR was used on all extracts using a Thermo Surveyor HPLC system coupled with an LTQ XL Linear Ion Trap Mass Spectrometer. Separation was achieved using the same column used in MMPB analysis (Kinetex C18 column) with mobile phase C (2 mM formic acid and 3.6 mM ammonium formate in deionized water) and D (95% acetonitrile (*v*/*v*) in 2 mM formic acid and 3.6 mM ammonium formate). Acetonitrile (Optima LC/MS), water (HPLC), ammonium formate (ACS grade), and formic acid (98%) were from Thermo Fisher Scientific (Waltham, MA, USA). The gradient (0.2 mL min^-1^) was as follows: solvent C 70–30% over 5 min, 30–70% C over 2 min, and held at 70% C for 3 min. Each chromatographic run was 10 min and 20 µL full loop injections were employed. The analysis of MC-LR (*m*/*z* 995.5→375.0, 553.4, 599.4, 866.6) was calibrated using a seven-point IS curve (0.5–100 ng mL^−1^) with *d*7-MC-LR (*m*/*z* 1002.5→599.5).

The analysis of the vomit extracts was conducted using targeted LC-MS/MS (Table S1) and LC-MS scans (*m/z* 400–1800 (-ve and +ve)) with a PDA set to λ 238 nm. The gradient (0.2 mL min^-1^) was as follows: solvent C held at 70% for 11 min, 70–30% C over 9 min, 30–70% C over 2 min, and held at 70% C for 5 min. XCalibur v 2.2 (Thermo Fisher Scientific, Waltham, MA, USA) was utilized for data processing. Standards used to calibrate the method included CRMs (NOD-R, MC-LR, MC-RR, [Dha^7^]MC-LR) and the RM (MC-RY) from the National Research Council Canada (Halifax, NS, Canada), RMs (MC-WR, [Asp^3^]MC-RR, [Asp^3^]MC-LR, MC-HtyR, MC-LF, MC-LW, MC-HilR) from Enzo Biochem (Farmingdale, NY, USA) and RMs ([Leu^1^]MC-LR, MC-YR, MC-LA, MC-LY) from GreenWater Laboratories (Palatka, FL, USA). Additional RMs ([ADMAdda^5^]MC-LR, [ADMAdda^5^]MC-LHar) were produced in-house through the extraction and purification of a strain of *Nostoc* sp. 152 generously supplied by Kaarina Sivonen (University of Helsinki) using methods previously described [38]. The desmethyl RMs [DMAdda^5^]MC-LR and [DMAdda^5^]MC-LHar were prepared by hydrolysis of the [ADMAdda^5^]MCs [57]. Quantification of targeted MCs was conducted using the previously reported IS approach [36].

## Figures and Tables

**Table 1:** Subjects examined in this study shown with weights, age, sex, date of exposure and status. UE = Unexposed Individual. N = neutered, S = spayed.

ID	Breed	Age (years)	Sex	Weight (kg)	Date of Exposure	Status
C-SP	Standard Poodle	9	Male/N	23	4 September 2018	Deceased
C-GR #1	Golden Retriever	6	Female/S	32	8 September 2018	Living
C-GR #2	Golden Retriever	2	Female/S	30	8 September 2018	Living
C-GR #3	Golden Retriever	4	Female/S	31	1 September 2018	Living
C-Pom	Pomeranian	2	Female/S	2.3	26 August 2018	Living
C-Chih	Chihuahua	6	Male/N	5.5	26 August 2018	Living
C-LR	Labrador Retriever	<1	Male	36	UE	Living
C-GD	Goldendoodle	10	Female/S	34	UE	Deceased
C-CBR	Chesapeake Bay Retriever	11	Female	34	UE	Living

**Table 2:** Clinicopathological abnormalities noted during hospitalization of six dogs exposed to the St. Lucie River HAB event. ID = Identification, APTT/PT = activated partial thromboplastin time/prothrombin time, ALT = Alanine aminotransferase, >DL = greater than detection limit.

ID:	C-SP	C-GR #1	C-GR #2	^1^C-GR #3	C-Pom	^1^C-Chih
Vomiting:	Yes	Yes	Yes	Yes	Yes	Yes
Melena:	Yes	No	No	Yes	Yes	No
Tachycardia:	Yes	No	No	Yes	No	Yes
Body cavity effusion:	Yes	Yes	No	Yes	Yes	Unknown
APTT/PT:	>DL	>DL	Normal	>DL	>DL	128/17
Thrombocytopenia:	12K	15K	60K	69K	24K	77K
Bilirubin (mg/dL):	2.1	15	0.1	3.1	2.2	7.2
ALT (U/L):	>DL	10K	1889	3294	5287	>DL
Blood Glucose (mg/dL):	26	74	97	66	27	120

**Table 3:** Results of the Adda MC/NOD analyses conducted on all matrices. Data is reported in parts per billion, with organ and hair samples reported by weight (ng g^−1^) and liquid samples by volume (ng mL^−1^). Data is reported ± the standard deviation for samples with duplicate extractions. MMPB analysis represents total Adda MCs, ELISA represents freely extracted Adda MCs and MC-LR is LC-MS/MS analysis of the freely extracted variant. PE = Post exposure. UE = Unexposed individual.

ID	# Days PE	Specimen	Total Adda MCs (MMPB)	Spike Return	Adda ELISA	Spike Return	MC-LR	Spike Return
C-SP	2	Liver	530	21%	260 ± 78	193%	7.2 ± 1.5	97%
	2	Kidney	2800	12%	1100 ± 460	106%	20 ± 7.2	63%
	2	Heart Blood	73	9%	85 ± 28	146%	1.7 ± 0.0	68%
	2	Bile	5400 ± 750	72%	7500 ± 3200	211%	65 ± 26	78%
	2	Urine	41,000	79%	42,000 ± 4000	143%	670	57%
C-GR #1	1	Blood	50	27%	―	―	―	―
	1	Urine	32,000 ± 1600	42%	22,000 ± 4500	197%	110 ± 26	―
	11	Urine	4.4	65%	―	―	―	―
	24	Urine	2.6 ± 1.0	35%	―	―	―	―
	68	Urine	0.6 ± 0.0	22%	―	―	―	―
	72	Hair	180 ± 19	4%	< 30	0%	< 30	111%
C-GR #2	1	Blood	70	74%	33	―	< 1.0	―
	0	Vomit	46,000 ± 8000	114%	25,000 ± 1800		14,000 ± 1700	
	10	Urine	0.6 ± 0.3	72%	―	―	―	―
	24	Urine	< 0.2	36%	―	―	―	―
C-Pom	25	Blood	< 0.2	8%	―	―	―	―
	23	Urine	2.6	37%	―	―	―	―
	37	Urine	0.2^1^	26%	―	―	―	―
	85	Urine	< 0.2	11%	―	―	―	―
C-CBR	UE	Hair	< 20	4%	< 30	133%	< 30	108%
C-GD	UE	Liver	< 4.0	7%	52^2^	33%	< 5	108%
C-LR	UE	Urine	< 0.2	36%	< 15	107%	< 2	79%
C-LR	UE	Blood	< 0.2	3%	< 15	92%	< 2	83%

^1^ above the limit of detection, but below the limit of quantification. ^2.^ false positive data (refer to text for explanation).

**Table 4:** Method detection limits (MDLs) achieved on each specimen type at lowest dilution analyzed. Reported as ng mL^−1^ unless otherwise specified.

Specimen	Total Adda MCs (MMPB)	Adda ELISA	MC-LR (LC-MS/MS)
Liver (ng g^−1^)	4.0	15	5.0
Kidney (ng g^−1^)	4.0	15	5.0
Bile	50	15	50
Blood	0.2	15	2.0
Urine	0.2	15	2.0
Hair (ng g^−1^)	20	30	30

## Supplementary Materials

The following are available online at https://www.mdpi.com/2072-6651/11/8/456/s1, Figure S1: A general representation of the heptapeptide microcystin and the formation of the MMPB molecule following oxidative cleavage of the Adda side chain; Figure S2: Significant gross lesions observed during autopsy of deceased dog (C-SP); Table S1: Multiple reaction monitoring (MRM) transitions for targeted analysis of MCs (19 variants), NOD-R; Table S2: Matrix spikes (of MC-LR) associated with returns reported in Table 3.
