# Supplementary material for: Diagnosing Microcystin Intoxication of Canines: Clinicopathological Indications, Pathological Characteristics, and Analytical Detection in Postmortem and Antemortem Samples
Source: Toxins (Basel). 2019 Aug 3;11(8):456. doi: 10.3390/toxins11080456 (PMC6722975; doi:10.3390/toxins11080456)
Supplement: Supplementary file 1 [file toxins-11-00456-s001.pdf]

Supplementary Materials: Diagnosing microcystin intoxication of canines: Clinicopathological indications, pathological characteristics, and analytical detection in postmortem and antemortem samples

Amanda J. Foss, Mark T. Aubel, Brandi Gallagher, Nancy Mettee, Amanda Miller and Susan B. Fogelson

Table S1. Multiple reaction monitoring (MRM) transitions for targeted analysis of MCs (19 variants), NOD-R and internal standards (*d*7-MC-LR, *d*5-MC-LF) used in LC-MS/MS Analyte.

	Precursor Ion (*m/z*)		Fragment Ions (*m/z*)	%CE
[DMAdda5]MC-LR	$[M+H]^+$	981.5	539.3, 553.4, 585.4, 599.4, 852.6, 953.6, 963.6	14%
[DMAdda5]MC-LHar	$[M+H]^+$	995.5	375.2, 553.4, 599.4, 866.6, 875.6, 967.6, 977.6	18%
[DAsp3]MC-RR	$[M+2H]^{2+}$	512.9	291.3, 426.3, 445.8, 503.8	28%
MC-RR	$[M+2H]^{2+}$	519.9	298.3, 440.3, 452.8, 455.3, 503.8	20%
NOD-R	$[M+H]^+$	825.5	389.4, 674.5, 691.5, 753.5, 781.5, 808.0	24%
MC-YR	$[M+H]^+$	1045.5	440.3, 599.4, 602.4, 1017.5, 1027.5	20%
MC-HtYR	$[M+H]^+$	1059.5	599.4, 634.4, 1031.6, 1041.6	16%
MC-LR	$[M+H]^+$	995.5	375.2, 553.4, 599.4, 866.6, 875.6, 967.6, 977.6	18%
[DAsp3]MC-LR	$[M+H]^+$	981.5	539.3, 553.4, 585.4, 599.4, 852.6, 953.6, 963.6	14%
[Dha7]MC-LR	$[M+H]^+$	981.5	539.3, 553.4, 585.4, 599.4, 852.6, 953.6, 963.6	14%
[ADMAdda5]MC-LR	$[M+H]^+$	1023.5	553.4, 627.5, 738.8, 963.6, 995.6, 1005.6	25%
MC-HilR	$[M+H]^+$	1009.5	567.4, 599.4	20%
[ADMAdda5]MC-LHar	$[M+H]^+$	1037.6	599.5, 613.4, 641.5, 908.6, 977.6, 1009.5, 1019.6	21%
MC-WR	$[M+H]^+$	1068.6	599.4, 626.3, 939.5, 1040.6	21%
[DLeu1]MC-LR	$[M+H]^+$	1037.6	599.5, 613.4, 641.5, 908.6, 977.6, 1009.5, 1019.6	21%
MC-RY	$[M-H]^-$	1043.5	1001.6, 1025.6	20%
MC-LA	$[M-H]^-$	908.5	780.0, 797.0, 878.0, 891.0	20%
MC-LY	$[M-H]^-$	1000.5	872.0, 889.0, 970.0, 983.0	20%
MC-LW	$[M-H]^-$	1023.6	1005.6	20%
MC-LF	$[M-H]^-$	984.76	966.6	20%
d7-MC-LR	$[M+H]^+$	1002.5	599.54	20%
d5-MC-LF	$[M-H]^-$	984.6	966.6	20%

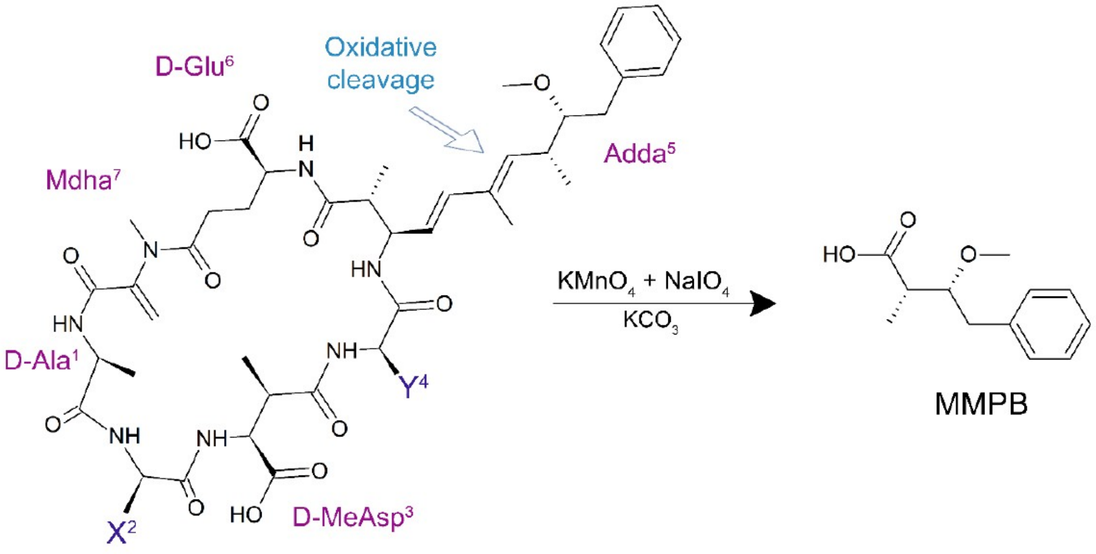

Figure S1. A general representation of the heptapeptide microcystin (left) and the formation of the MMPB molecule following oxidative cleavage of the Adda side chain. The amino acids at X2 and Y2 illustrate the sites of amino acid placement responsible for their principal variability and is represented using single letter amino acid code (e.g., L=leucine). Both intact microcystins and MMPB were analyzed in this work.
